# The Synergistic Priming Effect of Exogenous Salicylic Acid and H_2_O_2_ on Chilling Tolerance Enhancement during Maize (*Zea mays* L.) Seed Germination

**DOI:** 10.3389/fpls.2017.01153

**Published:** 2017-07-05

**Authors:** Zhan Li, Jungui Xu, Yue Gao, Chun Wang, Genyuan Guo, Ying Luo, Yutao Huang, Weimin Hu, Mohamed S. Sheteiwy, Yajing Guan, Jin Hu

**Affiliations:** ^1^Seed Science Center, Institute of Crop Science, College of Agriculture and Biotechnology, Zhejiang UniversityHangzhou, China; ^2^Department of Agronomy, Faculty of Agriculture, Mansoura UniversityMansoura, Egypt

**Keywords:** maize, seed priming, chilling tolerance, salicylic acid, H_2_O_2_, GA biosynthesis, ABA catabolism, antioxidant enzymes

## Abstract

Chilling stress is an important constraint for maize seedling establishment in the field. To examine the role of salicylic acid (SA) and hydrogen peroxide (H_2_O_2_) in response to chilling stress, we investigated the effects of seed priming with SA, H_2_O_2_, and SA+H_2_O_2_ combination on maize resistance under chilling stress (13°C). Priming with SA, H_2_O_2_, and especially SA+H_2_O_2_ shortened seed germination time and enhanced seed vigor and seedling growth as compared with hydropriming and non-priming treatments under low temperature. Meanwhile, SA+H_2_O_2_ priming notably increased the endogenous H_2_O_2_ and SA content, antioxidant enzymes activities and their corresponding genes *ZmPAL, ZmSOD4, ZmAPX2, ZmCAT2*, and *ZmGR* expression levels. The α-amylase activity was enhanced to mobilize starch to supply metabolites such as soluble sugar and energy for seed germination under chilling stress. In addition, the SA+H_2_O_2_ combination positively up-regulated expressions of gibberellic acid (GA) biosynthesis genes *ZmGA20ox1* and *ZmGA3ox2*, and down-regulated GA catabolism gene *ZmGA2ox1* expression; while it promoted GA signaling transduction genes expressions of *ZmGID1* and *ZmGID2* and decreased the level of seed germination inhibitor gene *ZmRGL2*. The abscisic acid (ABA) catabolism gene *ZmCYP707A2* and the expressions of *ZmCPK11* and *ZmSnRK2.1* encoding response receptors in ABA signaling pathway were all up-regulated. These results strongly suggested that priming with SA and H_2_O_2_ synergistically promoted hormones metabolism and signal transduction, and enhanced energy supply and antioxidant enzymes activities under chilling stress, which were closely relevant with chilling injury alleviation and chilling-tolerance improvement in maize seed.

**Highlights:**Seed germination and seedling growth were significantly improved under chilling stress by priming with SA+H_2_O_2_ combination, which was closely relevant with the change of reactive oxygen species, metabolites and energy supply, hormones metabolism and regulation.

## Introduction

Chilling stress is one of the most severe abiotic stresses inhibiting obviously seed germination and plant growth (Allen and Ort, 2001). Maize, one of sensitive crops to chilling stress (Hola et al., 2007), often encounters low temperature when sowed in early spring, resulting in reduction of seed emergence and seedling growth (Farooq et al., 2008b; Guan et al., 2009; Imran et al., 2013). Therefore, it is necessary to develop novel methods to enhance the chilling tolerance of maize seed (Maslak et al., 2007).

Hydrogen peroxide (H_2_O_2_) at proper concentration was conducive to seed dormancy-broken and germination enhancement (Müller et al., 2009; Lariguet et al., 2013). However, the over-accumulation of H_2_O_2_ easily caused cell injury (Mittler et al., 2004; Bailly et al., 2008; Kumar et al., 2015). H_2_O_2_ signal directly or indirectly involved in many signaling pathways of SA, GA, ABA, and so on (Durner and Klessig, 1999; Diaz-Vivancos et al., 2013; Maarouf et al., 2015; Qi et al., 2015). Antioxidant enzymes produced in plants such as superoxide dismutase (SOD), catalase (CAT), ascorbate peroxidase (APX), and glutathione reductase (GR) contributed to the endogenous H_2_O_2_ balance (Noctor and Foyer, 1998; Slesak et al., 2007). However, there are few reports on chilling resistance improvement of maize seeds through H_2_O_2_ application.

Salicylic acid (SA) has been well known as a signaling molecule in inducing the plant defensive system to biotic or abiotic stresses (Horvath et al., 2007; Rivas-San Vicente and Plasencia, 2011; Dong et al., 2014). Under low temperature, the enhanced biosynthesis of endogenous SA was closely relevant with the increasing antioxidant enzymes activities and seedling growth in maize seeds (Wang et al., 2013). Guan et al. (2015) reported that as seedling suffering from chilling stress (5°C), dry weight of roots and shoots, and protective enzymes activities in SA-treated coating maize seeds were obviously higher than those SA-untreated ones. Although SA was found to enhance chilling tolerance of maize, and H_2_O_2_ could promote seed germination, few reports concentrated on the combination effect of SA and H_2_O_2_ on maize chilling tolerance enhancement, and the mechanism involved in their combination effect remained completely unclear.

During maize seed germination, the α-amylase played an important role in starch decomposing into soluble sugar (Sun et al., 2015). In addition, seed germination was generally inhibited by high ABA concentration and promoted by GA in many species (Olszewski et al., 2002; Yamauchi et al., 2004). The dynamic balance of synthesis and catabolism of ABA and GA was crucial for seed germination (Footitt et al., 2011), The *NCED* encoding 9-*cis*-epoxycarotenoiddioxygen-ase was known as the key gene in ABA biosynthesis, and *CYP707A* encoding abscisic acid 8-hydroxylases as the essential gene in ABA catabolism (Footitt et al., 2011). ABA endogenous messenger genes *CPK11* and *SnRK2.1*, respectively, encoding calcium-dependent protein kinase and sucrose non-fermenting related protein kinase, were reported to be up-regulated under drought, salt and chilling stresses (Mao et al., 2010; Chen et al., 2012; Yang et al., 2014). The *GA20ox* (encoding GA 20-oxidase) and *GA3ox* (encoding GA 3-oxidase) were main genes during bioactive GA biosynthesis. In contrast, *GA2ox* encoding GA 2-oxidase antagonizes could active GAs to inactive GAs. Plant growth repressor DELLA proteins (DELLAs) consisting of GAI (GA-insensitive), RGA (Repressor of ga1-3) and RGL1-3 (RGA-like1-3) were inhibitors in GA signal pathway. In which, RGL2 was considered as the key repressor of seed germination (Stamm et al., 2012). The bond of GA with GID (gibberellin insensitive dwarf protein, a receptor of GA signaling) could trigger the degradation of RGL2, resulting in fast seed germination (Cao et al., 2006).

In this experiment, we found that maize seeds germinated obviously faster after SA+H_2_O_2_ combined priming as compared with priming treatment with SA or H_2_O_2_ alone. Therefore, antioxidant enzymes activities, α-amylase activities and corresponding genes expression involving in metabolism and signal transduction were determined after priming with SA, H_2_O_2_, and SA+H_2_O_2_ combination to acquire better understandings on the potential mechanism of SA+H_2_O_2_ priming in maize seed chilling tolerance enhancement.

## Materials and Methods

### Materials

Maize seeds of Meiyu No.3 with 10.1% moisture content were obtained from HaiNan Lv Chuan Seed Co., LTD. SA and H_2_O_2_ were purchased from Shanghai Dingguo Biotech Co., Ltd., Shanghai, China.

### Seed Priming

Seeds were surface sterilized with 0.5% NaClO solution for 15 min and then washed three times with sterilized distilled water to remove the residue of disinfectant. The sterilized seeds were primed with priming solutions (1:5, w/v) at 20°C in darkness for 24 h, without changing the priming solution during the process. Then all primed seeds were air-dried at 25°C for 48 h to their original moisture contents. In this study, four priming solutions including distilled water (hydropriming), 0.5 mM SA, 50 mM H_2_O_2_, and 0.5 mM SA+50 mM H_2_O_2_ were carried out.

### Seed Germination and Seedling Quality Measurement

All primed maize seeds were germinated in rolled towels moistened with water for 7 days in growth chambers at chilling stress (13°C), with a photosynthetic active photon flux density of 250 mmol m^-2^⋅s^-1^ and a photoperiod of 12 h light (L):12 h dark (D) (Wang et al., 2013). Those treatments were, respectively, indicated by HP+CS (hydropriming and chilling stress), SA+CS (SA priming and chilling stress), H_2_O_2_+CS (H_2_O_2_ priming and chilling stress), and SA+H_2_O_2_+CS (SA+H_2_O_2_ priming and chilling stress). Six replications of 50 seeds each for each treatment were used. The geminated seeds (5 mm radicle penetrated through seed coat) were counted daily for 7 days, and then germination energy (GE) and germination percentage (GP) was calculated on day 4 and day 7, respectively (ISTA, 2004). The germination index (GI) was measured as GI = Σ (Gt/Tt) (Hu et al., 2005). The mean germination time (MGT) was calculated as MGT = Σ (Gt × Tt)/ΣGt, where Gt is the number of new germinated seeds in time Tt. Shoot height and root length were measured manually with a ruler. The seedling dry weight (DW) was weighed directly after drying at 80°C for 24 h. Measurements were made on thirty randomly selected seedlings per replication. Vigor index (VI) was determined as VI = GI × DW (Hu et al., 2005).

### Hydrogen Peroxide, Antioxidant Enzymes, Malondialdehyde, α-Amylase, and Soluble Sugar Measurements

During seed germination after sowing, 10 seed embryos per replication and 6 replications for each treatment at each sampling time (0, 6, 24, 48, and 72 h after sowing) were collected for physiological parameters determination. The H_2_O_2_ content was measured according to the method of Doulis et al. (1997). The activities of SOD, POD, APX, and CAT were determined through the methods described by Qiu et al. (2005). POD, APX, and CAT activity was, respectively, calculated as μmol ascorbate decomposition min^-1^⋅g^-1^⋅FW and μmol H_2_O_2_ decomposition min^-1^⋅g^-1^⋅FW; while one unit of SOD activity was defined as 50% inhibition of nitro-blue tetrazolium (NBT) photochemical reaction and the results were expressed in U g^-1^⋅FW. GR was quantitated by measuring the decrease in absorbance at 340 nm due to the oxidation of nicotinamide adenine dinucleotide phosphate (NADPH), and GR activity was calculated as μmol NADPH oxidation min^-1^⋅g^-1^⋅FW (Smith and Johnson, 1988). The malondialdehyde (MDA) content was measured using the thiobarbituric acid reaction method (Gao et al., 2009). The activity of α-amylase was conducted using 3,5-dinitrosalicylic acid colorimetric method (Li et al., 2009). The soluble sugar content in seeds was measured by anthrone colorimetric method (Li and Li, 2013) with slight modification.

### Endogenous SA Measurements

Endogenous SA content was analyzed using HPLC according to the method of Wang et al. (2013). Each sample was ground into powder with liquid nitrogen and then 0.5 g of powder was homogenized in 1 ml aliquot of 90% (v/v) methanol. After centrifugation at 10,000 × *g* for 15 min, the supernatant was dried by nitrogen purging at 35°C. The residue was dissolved in 0.25 ml of 5% (w/v) TCA and the upper phase containing SA was concentrated by nitrogen purging at 35°C. In addition, the released SA was extracted from a lower aqueous phase. The SA collected from the upper phase and the lower phase were mixed and dissolved in a 600 μl of mobile phase consisting of 0.2 M sodium acetate buffer (pH 5.5) (90%) and methanol (10%), which was then filtered through a 0.22 μm membrane filter and analyzed by HPLC (Waters 600, Waters 717 automatic sampler, Waters, Milford, United States) under fluorescence detection (Waters 474). The excitation and emission wavelengths were 305 and 407 nm, respectively. Six replications of embryos for each treatment were collected at each sampling time.

### Total RNA Extraction and Quantitative Real-time PCR (RT-qPCR) Analysis

Total RNA was isolated from seed embryo using Trizol reagent (Huayueyang, Beijing, China) and reverse transcribed using a Rever Tra Ace qPCR RT kit (Toyobo, Osaka, Japan) following the manufacturer’s instructions. Gene specific RT-PCR primers were designed based on their cDNA sequences (Supplementary Table S1). Six genes involved in stress responses, i.e., *ZmSOD4, ZmAPX2, ZmCAT2*, and *ZmGR*. *ZmAMY* (α-amylase gene) involved in seed germination. Six genes involved in GA signal, i.e., *ZmGA20ox1, ZmGA3ox2, ZmGA2ox1, ZmGID1, ZmGID2*, and *ZmRGL2*. Four genes involved in ABA signal, i.e., *ZmNCED1, ZmCYP707A2, ZmCPK11*, and *ZmSnRK2.1*. *ZmPAL* (encoding phenylalanine ammonia-lyase) involved in SA biosynthesis. The RT-qPCR was performed using Roche real-time PCR detection system (Roche life science, United States). Each reaction (20 μL) consisted of 10 μL of SYBR Green PCR Master Mix (Takara, Chiga, Japan), 1 μL of diluted cDNA and 0.1 μM forward and reserve primers. The PCR cycling conditions were as follows: 95°C for 3 min, followed by 40 cycles of 95°C for 10 s and 58°C for 45 s. The maize Actin gene was used as an internal control. Relative gene expression was calculated according to Livak and Schmittgen (2001).

### Statistical Analysis

Data were analyzed by analysis of variance (ANOVA) using the Statistical Analysis System (SAS) (version 9.2) followed by calculation of the Least Significant Difference (LSD, *p* < 0.05). Percentage data were arc-sin-transformed prior to analysis.

## Results

### Seed Priming Enhanced Seed Germination Speed and Seedling Growth under Chilling Stress

**FIGURE 1:**
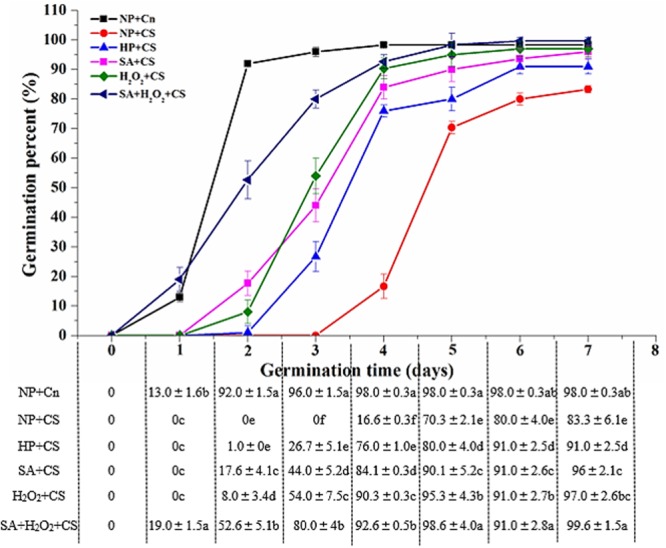
Seed priming enhanced seed germination speed under chilling stress (13°C). Vertical bars above mean indicated standard error of six replicates of 50 seeds each for each treatment. Values were mean ± SE (*n* = 6). Different small letter(s) following the values indicated significant difference (*p* < 0.05, LSD) among treatments. Other explanations were shown in **Table 1**.

As compared with NP+Cn, the seed germination was inhibited obviously under low temperature, and none of priming treatments reached the same germination speed and seedling growth compared to NP+Cn (**Figure 1**). The germination percentage of NP+Cn was 96% on the third day, when that of NP+CS was 0%. Seed priming treatments significantly promoted seed germination under chilling stress as compared with NP+CS. Seeds treated with SA+H_2_O_2_+CS started germinating at 1 day after sowing, whereas seeds treated with SA+CS or H_2_O_2_+CS began to germinate on the second day, while those seeds treated with HP+CS delayed germination until the third day after sowing (**Figure 1** and Supplementary Figure S2). On the third day, the maximum germination percentage (80.0%) was recorded in SA+H_2_O_2_ under chilling stress, as well as GE, GI, DW, and VI, which were also significantly higher than other priming treatments (**Table 1**). All priming treatments owned longer RL than NP+CS did, in which SA+H_2_O_2_ had the relatively higher level (6.05 cm). Priming treatments showed less of an effect on SH, in which H_2_O_2_ and SA+H_2_O_2_ significantly heighten SH compared to NP+CS (**Table 1**).

**Table 1:** Seed priming enhanced seed germination speed and seedling growth under chilling stress (13°C).

Treatments	GE (%)	GI	MGT (d)	SH (cm)	RL (cm)	DW (g/10plants)	VI
NP+Cn^∗^	98.0 ± 0.3a	54.4 ± 0.2a	1.94 ± 0.02a	4.64 ± 0.12a	7.77 ± 0.09a	1.0102 ± 0.0225a	57.3 ± 1.01a
NP+CS	16.6 ± 0.3f	16.9 ± 0.2c	4.98 ± 0.02f	1.05 ± 0.09d	2.11 ± 0.11f	0.0320 ± 0.0009d	0.55 ± 0.02d
HP+CS	76.0 ± 1.0c	24.4 ± 0.1bc	3.99 ± 0.01e	1.08 ± 0.10d	3.24 ± 0.08e	0.0569 ± 0.0018c	1.36 ± 0.04cd
SA+CS	84.1 ± 0.3d	20.8 ± 0.5bc	3.57 ± 0.03d	1.10 ± 0.09d	4.24 ± 0.09d	0.0670 ± 0.0018c	1.40 ± 0.04cd
H_2_O_2_+CS	90.3 ± 0.3c	29.6 ± 0.2b	3.45 ± 0.04c	1.30 ± 0.10c	5.16 ± 0.10c	0.0597 ± 0.0011c	1.77 ± 0.03c
SA+H_2_O_2_+CS	92.6 ± 0.5b	49.5 ± 0.3a	2.55 ± 0.05a	1.51 ± 0.11b	6.05 ± 0.06b	0.0889 ± 0.0004b	4.40 ± 0.02b

*^∗^Values were mean ± SE (*n* = 6). Different small letter (s) following the values indicated significant difference (*p* < 0.05, LSD) among treatments. The germination standard is 5 mm radicle penetrated through seed coat. GE, germination energy; GI, germination index; MGT, mean germination time; SH, shoot height; RL, root length; DW, the seedling dry weight; VI, vigor index. NP+Cn, no priming and no stress (25°C); NP+CS, no priming and chilling stress (13°C); HP+CS, hydropriming (water priming) and chilling stress; SA+CS, 0.5 mM salicylic acid priming and chilling stress; H_*2*_O_*2*_+CS, 50 mM hydrogen peroxide priming and chilling stress; SA+H_*2*_O_*2*_+CS, 0.5 mM salicylic acid and 50 mM hydrogen peroxide combination priming and chilling stress.*

### Seed Priming Increased Endogenous H_2_O_2_ and Decreased MDA Content under Chilling Stress

**FIGURE 2:**
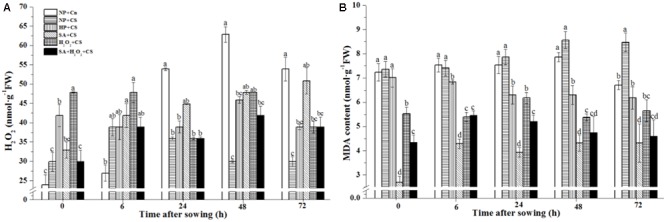
Seed priming increased endogenous H_2_O_2_ **(A)** and decreased MDA content **(B)** under chilling stress. Seed embryos were collected, respectively, at 0, 6, 24, 48, and 72 h after sowing, and six replications for each treatment at each sampling time were used. Different small letter(s) on the top of the bars indicated significant differences (*p* < 0.05, LSD) among treatments at the same time. Error bars indicated ± SE of mean (*n* = 6). For additional explanations, please see **Table 1**.

Hydrogen peroxide concentration gradually increased under normal condition (NP+Cn) and were significantly higher than NP+CS from 24 to 72 h after sowing (**Figure 2A**). The H_2_O_2_ content in NP+CS kept a stably low level and showed no visibly difference during seed germination (Supplementary Figure S1A). As comparing with NP+CS, the H_2_O_2_ concentration of H_2_O_2_+CS was significantly higher at 0 and 48 h; while SA+CS obviously improved H_2_O_2_ concentration from 48 to 72 h. SA+H_2_O_2_+CS enhanced H_2_O_2_ level in general, but did not show distinct difference with NP+CS and HP+CS from 6 to 72 h.

In addition, NP+Cn and NP+CS showed relatively higher MDA levels during seed germination than all priming treatments did (**Figure 2B** and Supplementary Figure S1B). The MDA content of NP+Cn decreased significantly at 72 h compared to NP+CS. Except for 24 and 72 h, HP had obviously higher MDA content than other three priming treatments. SA primed seeds showed the lowest MDA levels from 0 to 72 h, followed by SA+H_2_O_2_ and H_2_O_2_ primed seeds under chilling stress.

### Seed Priming Increased Endogenous SA Contents and Up-regulated ZmPAL Expression under Chilling Stress

**FIGURE 3:**
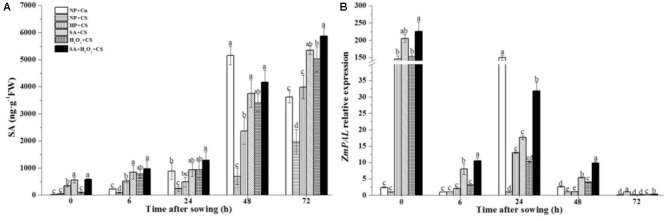
Seed priming increased endogenous SA contents **(A)** and up-regulated *ZmPAL* expression **(B)** under chilling stress. Seed embryos were collected, respectively, at 0, 6, 24, 48, and 72 h after sown, and six replications for each treatment at each sampling time were used. Different small letter(s) on the top of the bars indicated significant differences (*p* < 0.05, LSD) among treatments at the same time. Error bars indicated ± SE of mean (*n* = 6). For additional explanations, please see **Table 1**.

Non-primed seeds had prominently higher SA concentrations under 25°C than 13°C during seed germination (**Figure 3A**), and the notable increase in SA contents happened after 24 h after sowing. Under chilling stress, SA concentrations treated by SA+CS, H_2_O_2_ +CS, and SA+H_2_O_2_+CS rapidly increased as compared with NP+CS which were all significantly higher than HP+CS. All priming treatments increased SA contents markedly from 0 to 72 h under chilling stress.

The *ZmPAL* expression of NP+Cn reached its maximum level at 24 h after sowing (160-fold higher than NP+CS) (**Figure 3B**); while *ZmPAL* expression of NP+CS stayed at relatively low levels during 72 h after sowing. Under chilling stress, priming seeds significantly enhanced *ZmPAL* expression compared to NP+CS and NP+Cn at 0 h, and also up-regulated *ZmPAL* expression compared to NP+CS at 24 h. Z*mPAL* expression was obviously higher in case of SA+H_2_O_2_ combination than other three priming treatments from 6 to 48 h after sowing.

### Seed Priming Stimulated Antioxidant Enzymes Activities in Response to Chilling Stress

**FIGURE 4:**
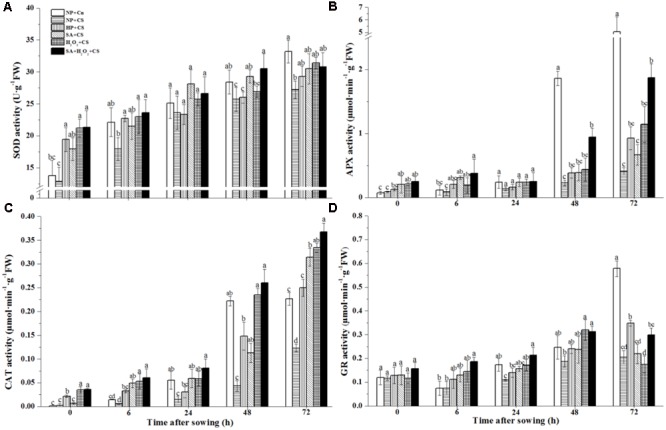
Seed priming stimulated antioxidant enzymes activities including superoxide dismutase (**A**, SOD), ascorbate peroxidase (**B**, APX), catalase (**C**, CAT) and glutathione reductase (**D**, GR) in response to chilling stress. Seed embryos were collected, respectively, at 0, 6, 24, 48 and 72 h after sowing, and six replications for each treatment at each sampling time were used. Different small letter(s) on the top of the bars indicated significant differences (*p* < 0.05, LSD) among treatments at the same time. Error bars indicated ± SE of mean (*n* = 6). For additional explanations, please see **Table 1**.

All of the antioxidant enzymes activities including SOD, APX, CAT, and GR of six treatments showed a stepped upward tendency during seed germination and reached highest levels at 72 h after sowing (**Figure 4**). These four enzymes activities of NP+CS were generally lower and had significant differences at 72 h as compared with those of NP+Cn. On the whole, all priming treatments improved four tested antioxidant enzymes activities compared to NP+CS, with a few exceptions such as HP+CS at 24 h in SOD and H_2_O_2_+CS at 72 h in GR. The relative higher levels of four antioxidant enzymes were all recorded in SA+H_2_O_2_ among four priming treatments under chilling stress.

### Seed Priming Enhanced *ZmSOD4, ZmAPX2, ZmCAT2*, and *ZmGR* Expression in Response to Chilling Stress

**FIGURE 5:**
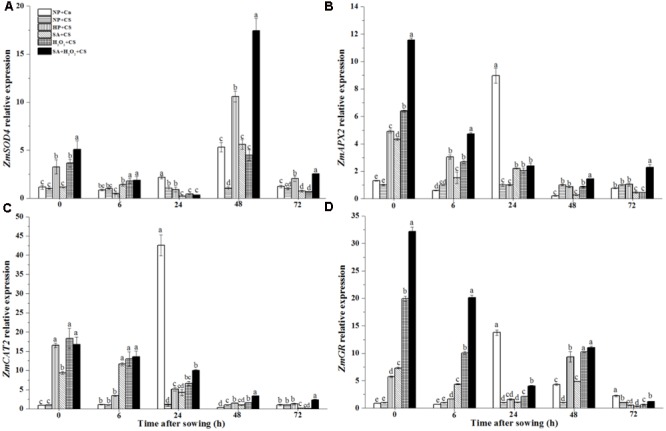
Seed priming enhanced *ZmSOD4* **(A)**, *ZmAPX2* **(B)**, *ZmCAT2* **(C)**, and *ZmGR* **(D)** expression in response to chilling stress. Seed embryos were collected, respectively, at 0, 6, 24, 48, and 72 h after sowing, and six replications for each treatment at each sampling time were used. Different small letter(s) on the top of the bars indicated significant differences (*p < 0.05*, LSD) among treatments at the same time. Error bars indicated ± SE of mean (*n* = 6). For additional explanations, please see **Table 1**.

The *ZmSOD4* expression of NP+Cn increased and got the highest level at 48 h, which was about six-fold higher than NP+CS (**Figure 5A**). At the end of seed priming, SA+H_2_O_2_+CS, H_2_O_2_+CS, and HP+CS significantly enhanced *ZmSOD4* expression compared to NP+Cn and NP+CS, and the higher *ZmSOD4* level was recorded in SA+H_2_O_2_. At 48 h, *ZmSOD4* expression in primed seeds increased fleetly, and SA+H_2_O_2_ had significantly higher *ZmSOD4* level than other treatments during seed germination except 6 and 24 h.

The expressions of *ZmCAT2 and ZmAPX2* in NP+Cn and NP+CS remained at low levels during seed germination after sowing; however, sudden increases happened in NP+Cn at 24 h, which were notably higher than those in NP+CS and priming treatments (**Figures 5B,C**). All four priming treatments improved *ZmCAT2 and ZmAPX2* expression compared with NP+Cn and NP+CS at 0 h, of which SA+H_2_O_2_+CS kept the higher levels during seed imbibition than other priming treatments.

The *ZmGR* expression of NP+Cn increased from 0 to 24 h after sowing, and the highest expression were recorded at 24 h in NP+Cn which was about 14-fold higher than NP+CS (**Figure 5D**). At the end of seed priming, the expression levels of *ZmGR* were significantly up-regulated, of which SA+H_2_O_2_ had the highest *ZmGR* level (32-fold higher than NP+CS) among the four priming treatments (**Figure 5D**, 0 h). In addition, *ZmGR* expression in SA+H_2_O_2_+CS was visibly higher than other three priming treatments from 0 to 24 h after sowing.

### Seed Priming Enhanced α-Amylase Activity and Soluble Sugar Content and Up-regulated *ZmAMY* Expression under Chilling Stress

**FIGURE 6:**
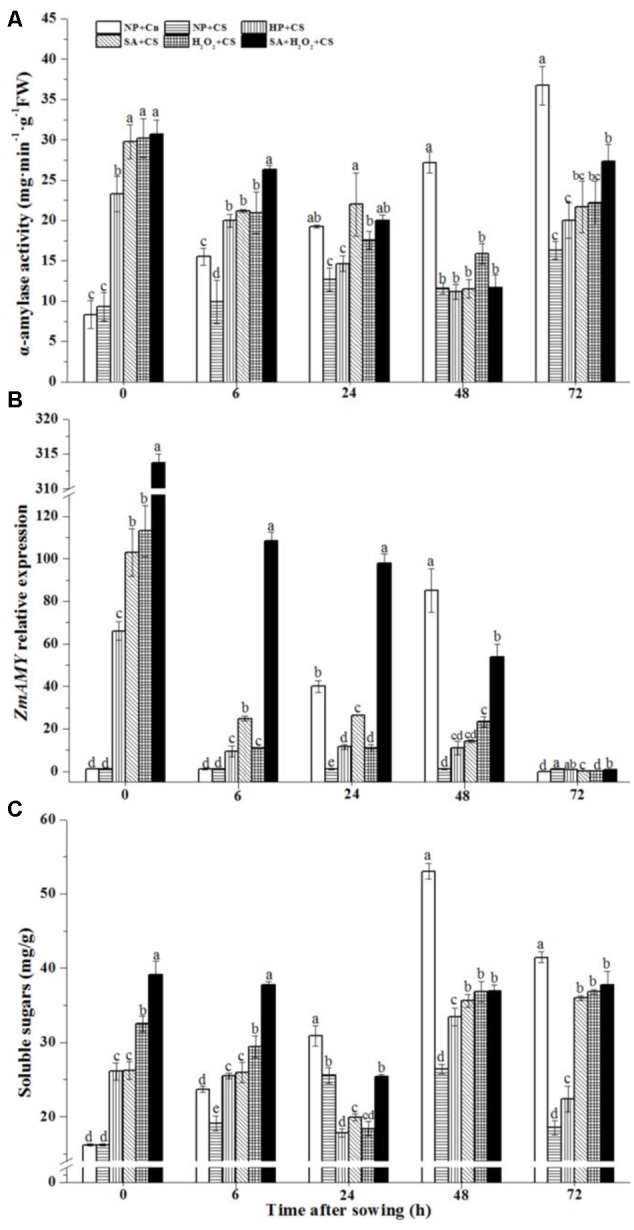
Seed priming enhanced α-amylase activity **(A)** and soluble sugar content **(C)** and up-regulated *ZmAMY* expression under **(B)** chilling stress. Seed embryos were collected, respectively, at 0, 6, 24, 48, and 72 h after sowing, and six replications for each treatment at each sampling time were used. Different small letter(s) on top of the bars indicated significant differences (*p < 0.05*, LSD) among treatments at same sown time. Error bars indicated ± SE of mean (*n* = 6). For additional explanations, please see **Table 1**.

The activity of α-amylase in NP+Cn increased gradually during seed germination, and reached higher level at 48 and 72 h than those in NP+CS and other priming treatments (**Figure 6A**). At the end of priming (0 h), α-amylase activities were improved obviously by primed treatments compared to those non-primed seeds, and seeds treated by SA, H_2_O_2_, and SA+H_2_O_2_ owned higher α-amylase levels than those treated by HP.

The expression of *ZmAMY* in NP+Cn was increased gradually from 0 to 48 h, and the highest value was recorded at 48 h which was about 90-fold higher than that in NP+CS (**Figure 6B**). *ZmAMY* expression were up-regulated significantly at the end of priming treatments (**Figure 6B**, 0 h), then sharply decreased from 0 to 6 h. SA+H_2_O_2_ remained *ZmAMY* expression above 100-fold more than NP+CS from 0 to 48 h. After sowing for 72 h, *ZmAMY* expression of all treatments fell back to a low level.

In addition, the soluble sugar content of NP+Cn increased rapidly to a maximum value at 48 h after sowing, and then accompanied by a slightly decline (**Figure 6C**). Except 24 h after sowing, priming treatments obviously improved soluble sugar content in maize seeds compared with NP+CS. During seed germination, SA+H_2_O_2_+CS kept a relatively higher level than other priming treatments except 48 h (**Figure 6C**).

### Seed Priming Regulated *ZmNCED1, ZmCYP707A2, ZmSnRK2.1*, and *ZmCPK11* Expression under Chilling Stress

**FIGURE 7:**
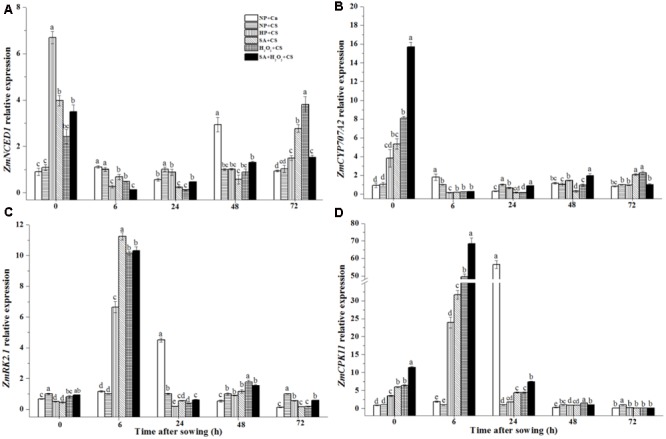
Seed priming regulated *ZmNCED1* **(A)**, *ZmCYP707A2* **(B)**, *ZmSnRK2.1* **(C)**, and *ZmCPK11* **(D)** expression under chilling stress. Seed embryos were collected respectively at 0, 6, 24, 48 and 72 h after sowing, and six replications for each treatment at each sampling time were used. Different small letter(s) on the top of the bars indicated significant differences (*p* < 0.05, LSD) among treatments at same sown time. Error bars indicated ± SE of mean (*n* = 6). For additional explanations, please see **Table 1**.

The *ZmNCED1* expression of NP+Cn had its relatively higher value at 48 h as compared with other times after sowing (**Figure 7A**). At the end of priming (0 h), four priming treatments increased obviously the *ZmNCED1* expressions than non-primed seeds, among which the highest level was recorded in HP. During seed germination from 0 to 6 h under chilling stress, *ZmNCED1* expression in priming treatments rapidly decreased; however, from 24 to 72 h, SA, H_2_O_2_, and SA+H_2_O_2_ presented a gradual upward trend.

*ZmCYP707A2* expressions of priming treatments were up-regulated significantly at 0 h as compared with NP+Cn and NP+CS (**Figure 7B**), in which SA+H_2_O_2_ up-regulated the expression of *ZmCYP707A2* by almost 16-fold than NP+CS. However, *ZmCYP707A2* expression decreased rapidly and stayed at low levels during time after sowing in different treatments.

The changing trends of *ZmSnRK2.1* and *ZmCPK11* expressions in four priming treatments were similar during seed germination (**Figures 7C,D**). At 6 h after sowing, they reached their highest levels, and*ZmSnRK2.1* expression in SA+CS, H_2_O_2_+CS, SA+H_2_O_2_+CS, and HP+CS were, respectively, 11-fold, 10-fold, 10-fold, and 6-fold higher than NP+CS; while *ZmCPK11* expression was, respectively, 33-fold, 50-fold, 65-fold, and 22.5-fold higher than NP+CS. In addition, *ZmSnRK2.1* and *ZmCPK11* expressions in NP+Cn reached maximum at 24 h, which was, respectively, 35-fold and 58-fold higher than NP+CS.

### Seed Priming Up-regulated *ZmGA20ox1, ZmGA3ox2, ZmRGL2, ZmGID1*, and *ZmGID2* Expression and Down-regulated *ZmGA2ox1* Expression under Chilling Stress

**FIGURE 8:**
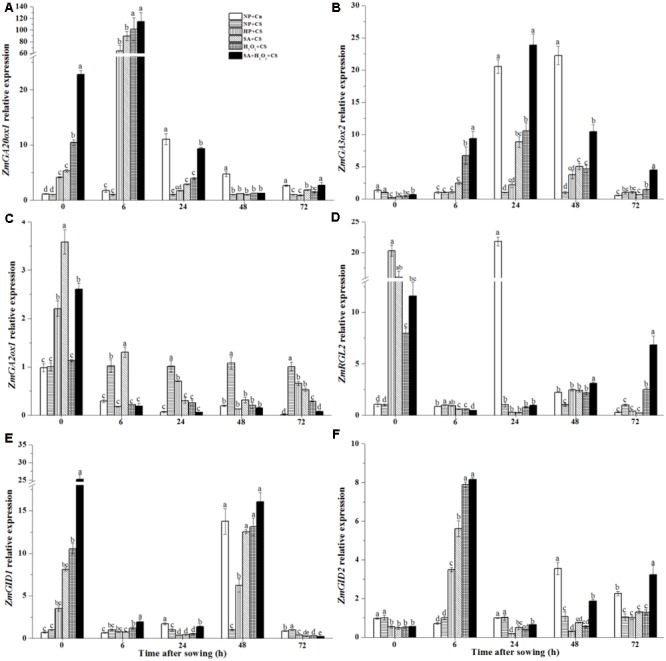
Seed priming up-regulated *ZmGA20ox1* **(A)**, *ZmGA3ox2* **(B)**, *ZmRGL2* **(D)**, *ZmGID1* **(E)** and *ZmGID2* **(F)** expression and down-regulated *ZmGA2ox1* **(C)** expression under chilling stress. Seed embryos were collected respectively at 0, 6, 24, 48, and 72 h after sowing, and six replications for each treatment at each sampling time were used. Different small letter(s) on the top of the bars indicated significant differences (*p* < 0.05, LSD) among treatments at the same time. Error bars indicated ± SE of mean (*n* = 6). For additional explanations, please see **Table 1**.

During seed germination, *ZmGA20ox1* and *ZmGA3ox2* genes were involved in GA biosynthesis. *ZmGA20ox1* expression of NP+Cn reached the highest level at 24 h (about 11-fold higher than NP+CS), and was significantly higher than NP+CS from 24 to 48 h (**Figure 8A**). At the end of priming treatments (0 h), the expression levels of *ZmGA20ox1* were up-regulated visibly. SA+H_2_O_2_+CS reached the highest *ZmGA20ox1* expression level (about 25-fold higher than NP+CS) which were distinctly higher than H_2_O_2_+CS, SA+CS, and HP+CS treatments. At 6 h after sowing, *ZmGA20ox1* expression remained increasing and reached obviously highest levels which were, respectively, 110-fold in SA+H_2_O_2_+CS, 100-fold in H_2_O_2_+CS, 90-fold in SA+CS, and 70-fold in HP+CS higher than NP+CS, and then they decreased rapidly.

The highest *ZmGA3ox2* expression of NP+Cn was recorded at 48 h, which was significantly higher than NP+CS and other treatments (**Figure 8B**). Four priming treatments up-regulated *ZmGA3ox2* expression compared with NP+CS. Seeds treated with SA, H_2_O_2_, and SA+H_2_O_2_ owned higher levels than those treated by HP from 6 to 48 h under chilling stress. And the significantly highest value was recorded in SA+H_2_O_2_+CS.

The *ZmGA2ox1* expression in NP+Cn was always significantly lower than that in NP+CS from 6 to 72 h (**Figure 8C**). Four priming treatments increased *ZmGA2ox1* expression after priming, which, however, decreased rapidly at the beginning of seed germination and significantly down-regulated as compared with NP+CS from 24 to 72 h. SA+H_2_O_2_+CS owned the lowest value among the four priming treatments.

The *ZmRGL2* expressions in NP+Cn and NP+CS stayed at low levels after priming (**Figure 8D**), the markedly highest level was showed at 24 h in NP+Cn which was about 22-fold higher than NP+CS and other treatments. The expression levels of *ZmRGL2* were significantly up-regulated at the end of priming treatments as compared with NP+CS, but then decreased rapidly.

The *ZmGID1* and *ZmGID2* genes are two positive regulators to mediate GA signaling pathways. The maximum levels of *ZmGID1* and *ZmGID2* expressions in NP+Cn were observed at 48 h, which were, respectively, about 13-fold and 3-fold higher than NP+CS (**Figures 8E,F**). After four priming treatments, *ZmGID1* and *ZmGID2* expressions were significantly up-regulated as compared with NP+Cn and NP+CS at 0 and 6 h, respectively. Seeds treated with SA+H_2_O_2_, H_2_O_2_, and SA had higher *ZmGID1* and *ZmGID2* expression values than those treated by HP (at 48 and 6 h, respectively) and the highest value was recorded in SA+H_2_O_2_ under chilling stress.

## Discussion

Chilling, one of the major abiotic stresses, exerts severe influences on growth and development of plant. In the present study, 13°C was applied as chilling temperature according to the report by Luo et al. (2015), and under chilling stress (NP+CS), germination of maize was delayed by ≥3 days after sowing compared with that in normal temperature (NP+Cn) (**Figure 1**). It was consistent with the results concluded by Farooq et al. (2008a) that the time to 50% emergence of hybrid maize was about 1.5 days shorter under normal condition than that under chilling stress. In comparison with the germination time of non-primed seed under 13°C (NP+CS), seeds primed with SA+H_2_O_2_ germinated 3 days in advance, seeds primed with SA or H_2_O_2_ germinated 2 days in advance and while seeds treated with HP germinated 1 day in advance (**Figure 1** and Supplementary Figure S2). Although the seedling quality of four priming treatments under chilling stress could not reach the NP+Cn level, they increased significantly the RL and DW compared to the NP+CS and among which SA+H_2_O_2_ had the highest values of SH, RL, DW, and VI. Obviously, priming treatments are useful (Elkoca et al., 2007; Pereira et al., 2009; Xu et al., 2011), which could be used for maize seeds germination acceleration and seedling quality improvement under chilling stress. Especially, SA and H_2_O_2_ had synergistical effect on seed germination enhancement.

The germinated ability of seeds seemed to be linked to the H_2_O_2_ accumulation to a critical level (Hossain et al., 2015; Huang et al., 2017). And H_2_O_2_ content was related with endosperm cap weakening and embryo elongation during lettuce seed germination (Zhang Y. et al., 2014). Our data showed that NP+Cn accumulated more H_2_O_2_ content from 24 to 72 h than NP+CS and other priming treatments; while four kind of priming seeds had relatively higher H_2_O_2_ levels than NP+CS from 48 to 72 h (**Figure 2**). However, over accumulation of H_2_O_2_ easily led to oxidative damage of membrane lipids, proteins, and nucleic acids (Das and Roychoudhury, 2014). The MDA content as an index of oxidative damage degree was consequently analyzed and the results showed that priming treatments obviously down-regulated MDA accumulation during seed germination under chilling stress. In addition, MDA concentrations in NP+CS and NP+Cn kept similarly high level from 0 to 24 h after sowing. However, with the extended germination time, MDA content in NP+Cn gradually decreased, while that in NP+CS continuously increased. It was suggested that the H_2_O_2_ accumulation in non-primed seeds under normal temperature or induced by priming under chilling stress did not cause an oxidative damage to seed embryo. However, NP+CS with the cumulative MDA under low temperature easily caused a harmful influence on the cell membrane integrity (Allen and Ort, 2001). Interestingly, priming seeds under chilling stress had even lower MDA content than non-primed seed under normal temperature, which might be due to seed membrane and organelle after priming had been repaired in advance, however, unprimed seed needed time to repair during seed germination.

Priming treatments significantly improved endogenous SA concentration during seed germination under chilling stress, which might be closely relevant with the up-regulated *ZmPAL* expression (**Figure 3**). Especially for SA+H_2_O_2_ combination, its SA content and *ZmPAL* expression level were obviously higher than those in SA or H_2_O_2_. Increased H_2_O_2_ and SA as signaling molecules could easily induce antioxidant defense system in plant (Mittler, 2002; Kang et al., 2003; Horvath et al., 2007). Comparing with NP+CS, the activities of antioxidant enzymes and their biosynthesis related genes expressions significantly enhanced after priming treatments (**Figures 4, 5**). Luo et al. (2011) also reported that the beneficial effect of SA on alleviating postharvest chilling injury of ‘Qingnai’ plum fruit closely related to the increased antioxidant enzymes activities and decreased MDA concentrations. Therefore, SA and H_2_O_2_ might synergistically promote activities of antioxidant enzymes to protect maize embryo against injury derived from low temperature.

During seed germination, starch in seed could be decomposed by α-amylase into maltodextrin, sucrose, glucose, etc. to provide metabolites and energy for seed germination and seedling growth, and so germination speed was highly associated with the starch metabolism (Hussain et al., 2015). The slower germination speed of maize seeds soaked at 10°C was closely related to the lower α-amylase activity as compared with seeds treated under 25°C (Silva-Neta et al., 2015). In this study, NP+Cn started to germinate at 24 h of after sowing, accompanied by obvious increase of α-amylase activity and its synthetic gene *ZmAMY* expression. However, chilling stress reduced a-amylase activity and total soluble sugars and seed reserves were not metabolized during the first 72 h after sowing, so non-primed seeds (NP+CS) did not germinate during this time. Therefore the rapidly increased α-amylase activity and enhanced soluble sugar content might be part of the reasons for the fast seed germination after priming treatments. It’s worth noting that at 24 h after sowing, the soluble sugar content had a sudden decrease in primed seeds, which might be the result of soluble sugars consumption by activated endogenous metabolism for germination preparation after priming. The *ZmAMY* expression involving in α-amylase synthesis were significantly up-regulated in priming treatments, even the SA+H_2_O_2_ improved the *ZmAMY* expression 113-fold higher than NP+CS. It indicated that priming treatments might enhance α-amylase activity though up-regulating *ZmAMY* expression under chilling stress. Seed germination was usually accompanied by the increases in ABA catabolism and GA biosynthesis (Liu et al., 2014; Zhang H. et al., 2014). The *cyp707a2* mutant, encoding the abscisic acid 80-hydroxylases, accumulated much more ABA and showed stronger dormancy than the wild type (Zhang H. et al., 2014). The over-expression of bean *PvNCED1* resulted in delayed seed germination and increased ABA content (Qin and Zeevaart, 2002). In our study, the expression levels of *ZmNCED1* and *ZmCYP707A2* increased significantly at the end of seed priming and then decreased rapidly during seed germination, which might contribute to the lower ABA concentration and faster germination in primed seeds than non-primed seeds under low temperature (**Figure 7**). The expressions of *ZmCPK11* and *ZmSnRK2.1* involving in ABA signal pathway notably up-regulated by seed priming compared with NP+CS under chilling stress. It was consistent with the results reported by Mao et al. (2010) that enhanced resistances to drought, salt and chilling were observed in *ZmCPK11* and *ZmSnRK2.1* over-expressing plants.

GA is another key regulator of seed germination in many species. A total of 136 different GAs have been identified in plants; however, only a few of GAs are biologically active (such as GA_1_ and GA_4_) and the predominantly active GA is usually species dependent (Olszewski et al., 2002; Yamauchi et al., 2004). Seeds from *GA3ox* deletion mutant could not germinate even under normal condition (Mitchum et al., 2006) and the transcript level of *GA3ox2* increased 40-fold in dormancy-broken seeds as compared with dormant ones (Finch-Savage et al., 2007). Whereas *GA2ox1* expression kept at obviously higher level in highly dormant seeds than in non-dormant seeds (Finch-Savage et al., 2007). After SA, H_2_O_2_, and SA+H_2_O_2_ priming, the expression levels of *ZmGA20ox1* and *ZmGA3ox2* involving in GA synthesis were up-regulated and those of *ZmGA2ox1* involving in GA catabolism were significantly down-regulated during maize seed imbibition at chilling condition (**Figure 8**). In addition, the expression of *ZmRGL2* involving in GA signal transduction was up-regulated at the end of priming, and then down-regulated sharply during germination. It was consistent with the model that GA improved germination by stimulating the SCF (SLY1) E3 ubiquitin ligase complex to trigger ubiquitination and destruction of RGL2 (Ariizumi and Steber, 2007). RGL2 protein was inferred as the inhibitor of seed germination (Lee et al., 2002). Ariizumi and Steber (2007) reported that seeds failing to germination in the GA biosynthesis mutant *ga1-3* could be rescued by exogenous GA application or by knocking out the DELLA gene *RGL2*. *ZmGID1* and *ZmGID2* positively regulated GA signaling transduction. The expression of *ZmGID1* was significantly up-regulated at 0 and 48 h, while those of *ZmGID2* significantly up-regulated at 6 h under chilling stress in this study. Therefore, enhanced GA signal transduction might be another reason for faster seed germination and better seedling establishment in primed seeds than non-primed seeds under chilling stress.

In addition, it is generally known that radicle breakout through seed coat and embryo eruption are two key points during seed germination (Dekkers et al., 2013). Radicle of maize seeds usually broke through seed coat after 24 h under normal temperature and embryo erupted after 48 h (**Figure 1**). Correspondingly, violently physiological and biochemical changes occurred usually during 24 to 48 h, such as GAs and ABA metabolism and signal transduction, antioxidant enzymes activities and α-amylase activities. As non-primed seed germinating at 13°C, radicle breakout delayed until 96 h of imbibition and embryo erupted happened until 7 days of germination, endogenous substances therefore had no dramatic changes during 72 h. However, the time for radicle breakout under low temperature predated to 24 h after seeds treated with SA+H_2_O_2_, the times predated to 48 h after seeds treated with SA or H_2_O_2_; while the time predated to 72 h after seeds treated with HP. Therefore, violent changes of endogenously physiological metabolism were correspondingly in advance at the end of priming.

**FIGURE 9:**
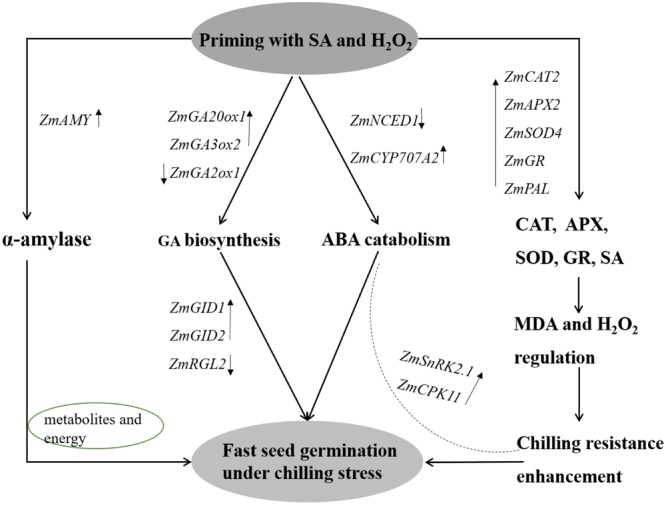
Proposed model of priming by SA and H_2_O_2_ on chilling tolerance improvement during maize (*Zea mays* L.) seed germination. The α-amylase was enhanced to decompose starch to provide metabolisms and energy for seed germination under chilling stress. Priming seeds with SA and H_2_O_2_ positively up-regulated GA biosynthesis and ABA catabolism, and promoted GA signaling transduction. SA content and antioxidant enzymes activities increased to alleviate chilling stress. *ZmCPK11* and *ZmSnRK2.1* expressions were up-regulated to improve the tolerance to chilling stress. SA, salicylic acid, H_2_O_2_, hydrogen peroxide.

Seed vigor and seedling establishment under chilling stress were notably improved in SA+H_2_O_2_ combination in tested maize seeds. There might underline several possible mechanisms (**Figure 9**). (1) Changes of reactive oxygen species: endogenous H_2_O_2_ and SA content increased and antioxidant enzymes activities enhanced after seed priming; (2) metabolism and energy supply: the enhancement of α-amylase activity indicated rapid mobilization of starch in seeds, which supplied metabolites and energy for seed germination and seedling growth under chilling stress; (3) hormones metabolism and regulation: GA biosynthesis genes *ZmGA20ox1* and *ZmGA3ox2* up-regulated, GA catabolism gene *ZmGA2ox1* and ABA biosynthesis gene *ZmNCED1* down-regulated. Meanwhile, GA signaling transduction genes *ZmGID1* and *ZmGID2* expression increased and seed germination inhibitor gene *ZmRGL2* level decreased after seed priming. In addition, the expression of *ZmCPK11* and *ZmSnRK2.1* encoding response receptors in ABA signaling pathway were up-regulated.

## Conclusion

Chilling tolerance of maize seeds could be significantly improved by SA+H_2_O_2_, which was obviously superior to SA or H_2_O_2_. However, the effect of SA+H_2_O_2_ on chilling-resistance improvement was not the simple addition of single effects of SA and H_2_O_2_. It definitely indicated that, based on the physiological and molecular data, SA and H_2_O_2_ could mutually induce and maintain homeostasis to exert synergistic effect on maize seeds chilling resistance. Furthermore, the underlined synergies of SA and H_2_O_2_ suggested that there were different mechanisms in chilling resistance induction by SA or H_2_O_2_; however, there existed interaction effects among different physiological and molecular mechanisms under chilling stress, which still needed further study.

## Author Contributions

Conceived and designed the experiments: ZL and YaG. Performed the experiments: ZL, JX, WH, and CW. Analyzed the data: ZL, YuG, MS. Contributed reagents/materials/analysis tools: JH, YL, YH, GG, and WH. Wrote the paper: ZL and YaG.

## Conflict of Interest Statement

The authors declare that the research was conducted in the absence of any commercial or financial relationships that could be construed as a potential conflict of interest.
