# Supplementary material for: The Synergistic Priming Effect of Exogenous Salicylic Acid and H2O2 on Chilling Tolerance Enhancement during Maize (Zea mays L.) Seed Germination
Source: Front Plant Sci. 2017 Jul 5;8:1153. doi: 10.3389/fpls.2017.01153 (PMC5496956; doi:10.3389/fpls.2017.01153)
Supplement: Supplementary file 1 [file Presentation_1.PDF]

The synergistic priming effect of exogenous salicylic acid and H_2O_2 on chilling tolerance enhancement during maize (*Zea mays* L.) seed germination

Zhan Li[1], Jungui Xu[1], Yue Gao[1], Chun Wang[1], Genyuan Guo[1], Yutao Huang[1], Weimin Hu[1], Mohamed Salah Sheteiwy[1,2], Yajing Guan[1*], Jin Hu[1]

[1] Seed Science Center, College of Agriculture and Biotechnology, Zhejiang University, Hangzhou 310058, China

[2]Department of Agronomy, Faculty of Agriculture, Mansoura University, Mansoura 35516, Egypt

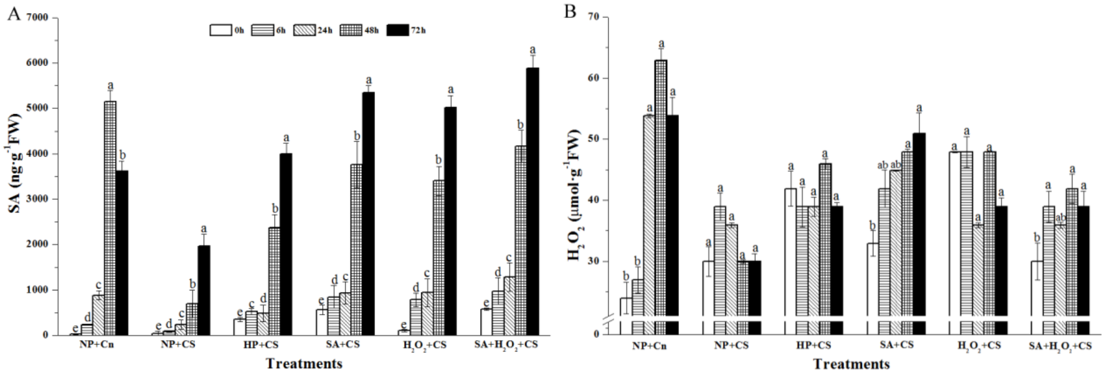

Fig. S1. Effects of seed priming on SA (A) and H_2O_2 (B) content in maize embryos after sowing under chilling stress (13 ℃). Seed embryos were collected respectively at 0, 6, 24, 48 and 72 h after sowing, and six replications for each treatment at each sampling time were used. Different small letter (s) on the top of the bars indicated significant differences ($p<0.05$, LSD) among different germination times at the same treatment. Error bars indicated ± SE of mean ($n = 6$). Other explanations were shown in Table 1.

*Corresponding author. Tel.: +86 57188982318; fax: +86 57188982318; E-mail address: vcguan@zju.edu.cn

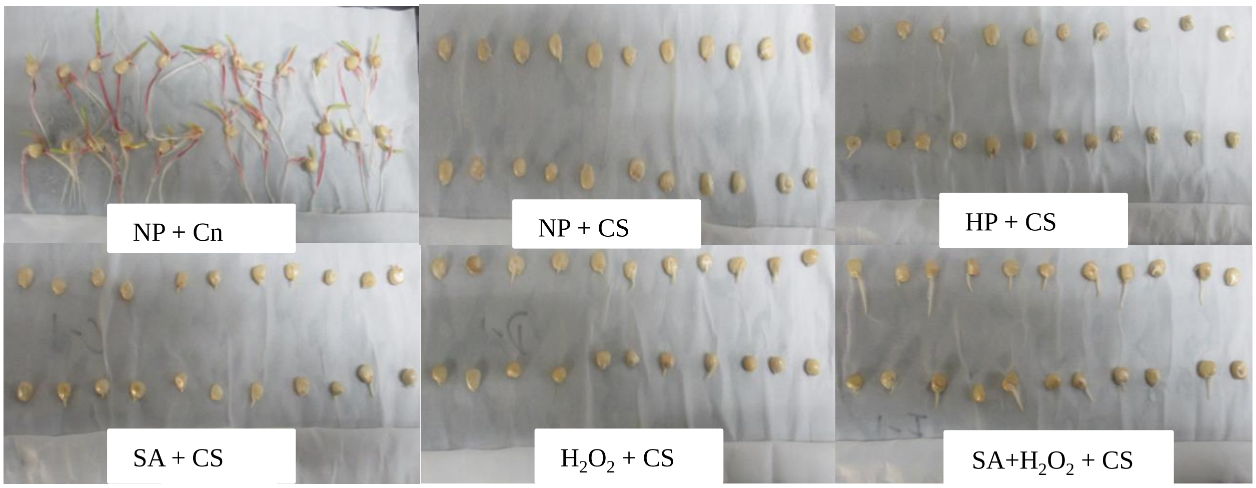

Fig. S2. Seed germination of different treatments on the third day after sowing. NP+Cn: no priming and no stress (25 ℃); NP+CS: no priming and chilling stress (13 ℃); HP+CS: hydropriming (water priming) and chilling stress; SA+CS: 0.5 mM salicylic acid priming and chilling stress; H_2O_2+CS: 50 mM hydrogen peroxide priming and chilling stress; SA+H_2O_2+CS: 0.5 mM salicylic acid and 50 mM hydrogen peroxide combination priming and chilling stress.

Table S1. Real-time PCR primers used for genes expression analysis

Gene Name	Forward	Reverse
ZmMAPK4	CAACCCCTGTTTCCTGGAC	TCATCTGGAGAGCCTATGAGC
ZmMAPK6	CCTACGGCATCGTCTGCT	GCATCGATCTTGTTGTCGAA
ZmAPX2	CCCATCCTATCCTACGCTGA	ATCAGGTCCGCCGGTTAC
ZmSOD4	GATCTTGGAAAGGGTGGACA	GAAGTCCAGCGACCCATTT
ZmCAT2	ATCAGGACCATCTGGCTCTC	GCGCCCTTTGTCTGGTAG
ZmGR	CAAGATTTTGATGCCACCATT	TTTTCTAGTCGGGCTCCTCA
ZmAMY	GGCACCACTAGACTAGACCCATT	GGTTCAAAATGGACCTGAAAAG
ZmGA20ox1	GGAGATGGACAAGGTGGTCA	ACGTGAAGTCCGGGTACG
ZmGA3ox2	ACGACGACGATCATCAGGTA	AGGTGAAGAAGCCCGAGTC
ZmGA2ox1	CTCACTCCTGCGGATAAACC	TCGTGGTCAAGCTTGTGAAT
ZmGID1	TGGTGGACTGATGGACCAA	CCCATCCGATCCATAAAAGA
ZmGID2	ACTGCTTATCGCACGTCCA	CATCAGAGCGGCACTTCAT
ZmRGL2	TATGCTGTTGGCGTTGTTTG	AGACACCCATCGTTCTCTTCA
ZmNCED1	CCCAAATCATCCATCACATTC	GGAGGATGGACTCTCCACTG
ZmCYP707A2	GGGAAGTACAACGAAGGCACT	TGCAACTTGTAAGAAGAGTGATCC
ZmCPK11	CTAAGATGCTGGACAATTTTCGT	CAGCCATCCCTCTTCTCTAGC
ZmSnRK2.1	GCAAATATGCCACCGAGATT	GACTTAGAATAACCAAAATCGCAAA
ZmPAL	ACATCTACGGCGTCACCAC	AAGATTCCGGCGTTGAGA
ZmActin	CGTTACCGGCTCATTG	AGGGAGCACCAGACTCAT
